# Endocrine disruptors in blue mussels and sediments from the Gulf of Gdańsk (Southern Baltic)

**DOI:** 10.1007/s11356-016-6524-5

**Published:** 2016-04-01

**Authors:** Anna Filipkowska, Ludwik Lubecki

**Affiliations:** Marine Pollution Laboratory, Institute of Oceanology, Polish Academy of Sciences, ul. Powstańców Warszawy 55, 81-712 Sopot, Poland

**Keywords:** Nonylphenols, Tributyltin, Organotins, Endocrine disruptors, Sediment, Mussels, Baltic Sea

## Abstract

Samples of blue mussel (*Mytilus trossulus*) and sediment were collected in the Gulf of Gdańsk (Southern Baltic Sea) to assess the extent of their contamination with two groups of endocrine disruptors: 4-nonylphenols and organotins (butyl- and phenyltins). Five sampling stations were chosen along the coastline of the Tricity Agglomeration (Gdańsk, Sopot, Gdynia) in 2008, 2012, and 2013. No evident differences between the three campaigns were found in either the mussel or the sediment samples. The mussels were moderately contaminated with 4-nonylphenols (30–111 ng g^−1^ d.w.), whereas the levels of these compounds in the sediment samples were very low (0.8–2.7 ng g^−1^ d.w.). Total concentrations of butyltin compounds in the mussels and sediments ranged between 41 and 164 ng Sn g^−1^ d.w., and from below the limit of detection to 22 ng Sn g^−1^ d.w., respectively, whereas phenyltins were not detected in any of the samples. Butyltin degradation indices indicate an old tributyltin input into the coastal environment, which is characterized by intense maritime activity. The results obtained from this work demonstrate that 5 years after the total ban on using organotin-based antifouling paints was imposed, butyltins are still present in mussels and sediments of the Gulf of Gdańsk.

## Introduction

Endocrine disruptors are chemical compounds of great environmental concern. They are a large class of substances of natural and/or anthropogenic origin that interfere with the hormone (endocrine) system of organisms, which may result in sterility and species extinctions as a consequence of reproductive and developmental disorders. A wide range of substances can disrupt the endocrine system: they include dioxin and dioxin-like compounds, polychlorinated biphenyls (PCBs), bisphenol A (BPA), polybrominated diphenyl ethers (PBDEs), DDT, phthalates, and pharmaceuticals (Diamanti-Kandarakis et al. 2009). In the marine environment, 4-nonylphenols (4-NPs) and organotins (OTs) (tributyltin (TBT) and triphenyltin (TPhT)) are also giving cause for concern. The last two groups of compounds exhibit different mechanisms of endocrine disruption: 4-NPs can mimic natural estrogens (feminization) (David et al. 2009; Soares et al. 2008), while OTs are androgenic compounds (masculinization) (Antizar-Ladislao 2008; Axiak et al. 2003).

4-NPs present in the environment are derived mainly from the breakdown of nonylphenol ethoxylates by the loss of ethoxy groups (Soares et al. 2008). Nonylphenol ethoxylates are highly efficient and cost-effective non-ionic surfactants and have therefore been widely used as ingredients of detergents, emulsifiers, solubilizers, wetting agents, and dispersants (David et al. 2009; EU 2002). Effluent discharges from municipal and industrial waste water treatment plants are the main sources of 4-NPs in aquatic environments (HELCOM 2009). Because of their hydrophobic properties, 4-NPs tend to become associated with suspended particles and are ultimately deposited in bottom sediments (Huang et al. 2007). 4-NPs may persist for decades in anaerobic environments (Diehl et al. 2012), while those deposited in recent sediments may be subject to resuspension, as well as biotic and abiotic transformations. Thus, they continue to pose a serious threat to marine organisms (de Weert et al. 2010). 4-NPs can be accumulated by aquatic organisms, especially in tissues with a high lipid content (EPA 2005). These compounds may have adverse effects on aquatic organisms: their estrogenic activity may cause endocrine-related malfunctions in biota (David et al. 2009). As endocrine disruptors, 4-NPs may cause feminization of aquatic organisms as well as decrease male fertility and reduce juvenile survival (Soares et al. 2008). These compounds therefore represent an ecological hazard at the population level. Because of their toxicological effects, persistence, and widespread usage, a drastic reduction policy in their use has been implemented in the European Union since January 2005 (EU 2003a).

TBT and TPhT are anthropogenic compounds that have been used for many years as antifouling ingredients in paints to prevent the settlement and growth of aquatic organisms on ship hulls, fishing nets or cages, oil rig supports, and different tools used in seawater. That is why considerable amounts of OTs have been released into the marine environment. Owing to their high toxicity, a total ban on the use of harmful OTs in antifouling paints was introduced in 2008 (EU 2003b; IMO 2001). However, these compounds still give cause for concern because of their environmental persistence, as well as their ability to be transferred along trophic chains (Ciesielski et al. 2004; Strand and Jacobsen 2005; Veltman et al. 2006). Even if TBT and TPhT are currently not released into the marine environment by vessels, harmful OTs deposited in sediments are still bioavailable as a result of their resuspension, diffusion into the water column, or a variety of transformations. The degradation processes in sediments depend on the environmental conditions and may last for years (the half-life of TBT is estimated at between a few months and several dozen years) (Dowson et al. 1996; Filipkowska et al. 2014; Takeuchi et al. 2004). TBT and TPhT can be harmful to various aquatic organisms (in particular to mollusks) at very low concentrations (e.g., even less than 1 ng TBT L^−1^ can cause imposex in marine snails). They cause various symptoms: the appearance of male characters in the female, reproductive disorders in mollusks (*Gastropoda* and *Bivalvia*), growth retardation in mussels, shell calcification anomalies in oysters, and immunological dysfunction in fish (Antizar-Ladislao 2008; Alzieu 2000; Hoch 2001; Rüdel 2003).

The problem of the presence of endocrine disruptors in the marine environment has been highlighted by many international commissions, e.g., HELCOM (*Baltic Marine Environment Protection Commission*), OSPAR (*Commission for the Protection of the Marine Environment of the North*-*East Atlantic*), and BSC (*Commission on the Protection of the Black Sea Against Pollution*). Moreover, both NPs and OTs are recognized as priority hazardous substances in the EU directive on environmental quality standards in the water framework policy (EU 2008). As far as the Baltic Sea is concerned, HELCOM has classified NPs and OTs as hazardous substances of specific concern and emphasized that the available studies of these compounds are insufficient (HELCOM 2010). Published data concerning the distribution and fate of studied xenobiotics in the Southern Baltic Sea are very scarce, and HELCOM strongly recommends these harmful substances to be monitored in different compartments of the environment, including bottom sediments and mussels. Bottom sediments are regarded as the final repository (sink) for hydrophobic chemicals, including 4-NPs and OTs, in the marine environment, while mussels are currently used as sentinel organisms of these pollutants (Albalat et al. 2002; Bortoli et al. 2003; Galassi et al. 2008; Staniszewska et al. 2014). Marine bivalves—including the genus *Mytilus*—are useful bioindicators in pollution monitoring programs, due to the fact they are (a) filter-feeders and therefore exposed to large volumes of seawater and (b) immobile and therefore exposed to local contamination only, and they have shown (c) limited ability to metabolise 4-NPs and TBT and (d) ability to accumulate and retain these compounds for a significant period, in direct proportion to the environmental levels (Chandrinou et al. 2007; David et al. 2009; Laughlin and French 1988; Morcillo et al. 1997; Mzoughi et al. 2005).

The aim of this work was to assess the extent of contamination by 4-NPs and OTs (tributyltin, triphenyltin, and their mono- and di-substituted degradation products) in sediments from the coastal area of the Gulf of Gdańsk, as well as in mussels *Mytilus trossulus*, which are used as bioindicators of organic pollution and are common enough to be collected with a drag in this area. An additional value of these OT studies is the fact that we analyzed samples collected just after the implementation of the total ban on OTs in antifouling paints and then—to assess the effectiveness of the regulation in this region—a few years after the total ban came into force. It is essential to monitor the fate of NPs and OTs in the marine environment since they can pose a serious threat to aquatic organisms, even at very low concentrations.

## Material and methods

### Site description and sample collection

**Fig. 1:**
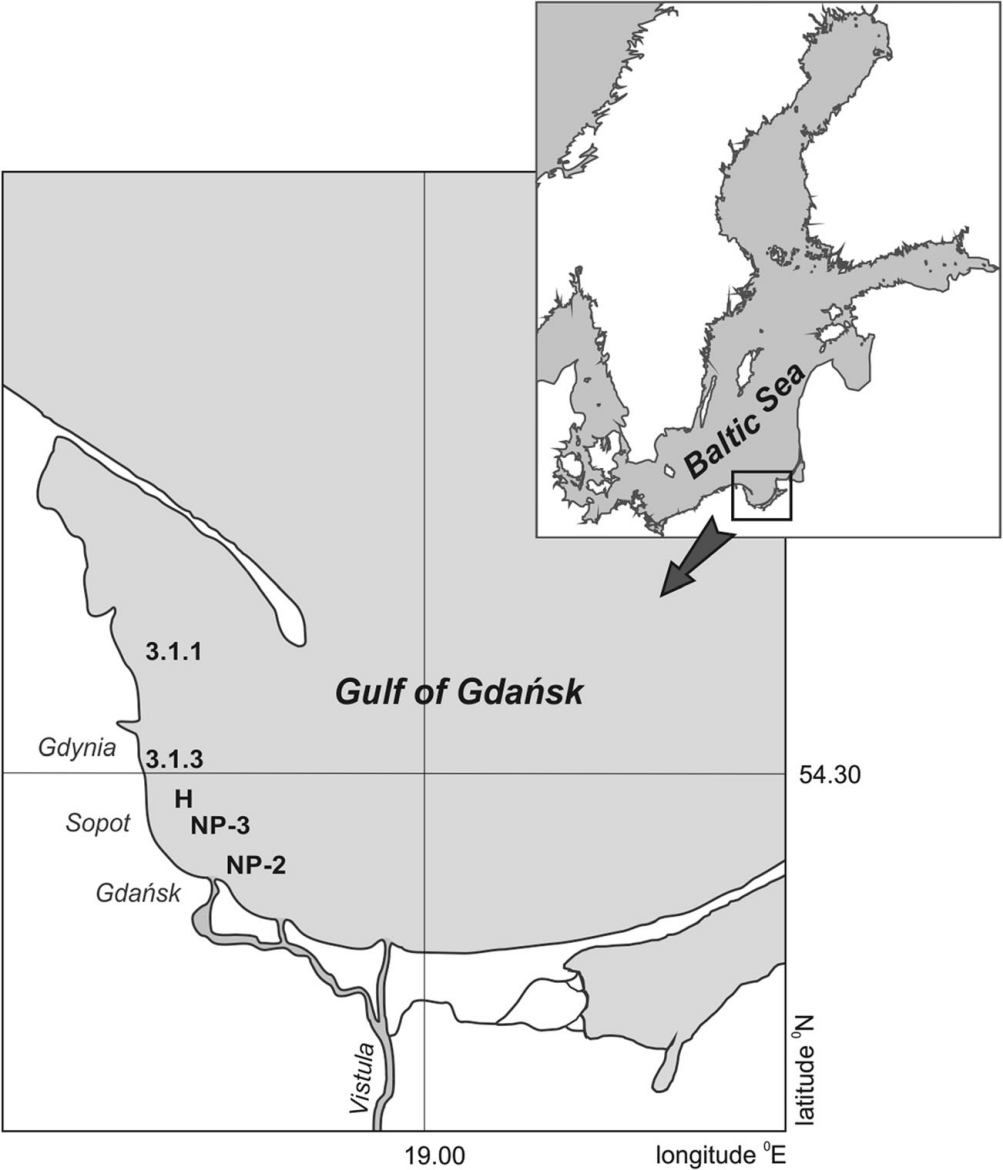
Location of the sampling stations

Samples of blue mussel (*Mytilus trossulus*) and sediment were collected in the western part of the Gulf of Gdańsk (Poland) (Fig. 1). The Gulf of Gdańsk (4940 km^2^) is located in the southeastern part of the Baltic Sea. As a result of the large inflows of fresh water from the Vistula River, the occasional inflows of North Sea water through the Danish Straits, as well as urban agglomerations and big international seaports in the immediate vicinity, this is a special environment and an interesting area for studying the fate of pollutants in the marine environment (Lubecki and Kowalewska 2010).

The samples were collected in 2008, 2012, and 2013, at five shallow stations (depth 12–15 m), located along the coastline of the Tricity Agglomeration (Gdańsk, Sopot, Gdynia) and given the preestablished site coding of 3.1.3, H, NP-3, 3.1.1, and NP-2 (Table 1). These sampling sites are potential habitats of blue mussels *Mytilus trossulus*, characterized by organic-poor sandy sediments, good oxygen conditions in the near-bottom water, and low salinity (7.0-7.4 PSU). Depth, temperature, salinity, and dissolved oxygen content in the near-bottom seawater were measured at each sampling station using a portable field meter (ProfiLine Multi 197i; WTW, Germany). In 2012 and 2013, the sampling could not be repeated at all the stations for lack of blue mussels.

**Table 1:** Description of the sampling stations and environmental conditions

Station	Coordinates	Location	Depth [m]	Salinity [PSU]	Oxygen [mg L^−1^]	Sampling campaign
3.1.3	54° 30′ 47″ N	Near the Port of Gdynia	12	7.0–7.4	9.0–10.8	2008, 2012, 2013
	18° 35′ 12″ E					
H	54° 29′ 08″ N	Near the Orłowo Cliff	13	7.0–7.4	9.1–10.9	2008, 2012, 2013
	18° 37′ 05″ E					
NP-3	54° 26′ 59″ N	Near the anchorage—Port of Gdańsk	12	7.0–7.3	9.1–10.8	2008, 2012, 2013
	18° 39′ 51″ E					
3.1.1	54° 36′ 29″ N	Near the Rewa Headland	15	7.1–7.3	9.1–11.0	2008, 2013
	18° 34′ 46″ E					
NP-2	54° 25′ 30″ N	Near the Port of Gdańsk	12	7.0–7.3	8.7–10.7	2008
	18° 43′ 05″ E					

The sediment samples (0–5 cm) were collected with a van Veen grab sampler, and the mussels with a drag. Following collection and depuration in clean seawater, the mussels were initially frozen, after which the soft tissues were separated from the shells (only individuals 20–50 mm long were selected). Both the mussel and sediment samples were stored at −20 °C, then freeze-dried and homogenized. The concentrations of NPs and OTs were determined in sub-samples.

### 4-Nonylphenol determinations

4-Nonylphenols were extracted from sediment samples according to the procedure described by Lubecki (2014); this procedure was modified slightly for the analysis of mussels. The samples were extracted by ultrasonication (3 × 15 min) in an acetone–n-hexane mixture (1:1 *v*/*v*, mussels 2 × 20 mL + 10 mL, sediments 3 × 20 mL). After centrifuging, the extracts were combined and preconcentrated using a rotary evaporator and then evaporated to dryness under a stream of argon. Next, the raw extract was cleaned up in sequence on two SPE cartridges: the first was packed with silica gel (70–230 mesh), the second with florisil (100–200 mesh). Each SPE column was conditioned with n-hexane. Next, the n-hexane solution of extract was added, and after flushing the bed with n-hexane and an n-hexane–dichloromethane mixture (1:1 *v*/*v*), the fraction containing 4-NPs was eluted with dichloromethane. The analytes were not derivatized. The nonylphenol extracts were analyzed using a gas chromatograph coupled to a mass spectrometer detector (Shimadzu GCMS-QP2010 Ultra). The extract dissolved in isooctane was injected in splitless mode. The injector was held at 300 °C. The carrier gas was helium at a flow rate of 1 mL min^−1^. A fused silica capillary column (CP-Sil 8 CB Low Bleed/MS, Varian) with a 5 % phenyl-substituted dimethylpolysiloxane phase (0.25 mm i.d. × 30 m, 0.25 μm film thickness) was used. The oven temperature was held isothermally at 80 °C for 2 min, then programmed at 30 °C min^−1^ to 200 °C, held for 2 min, and then increased by 10 °C min^−1^ to 280 °C and held for 5 min. The interface temperature was 280 °C. The detector (ion source 220 °C) was operated in electron impact ionization mode, and the emission current was 60 μA. Selected ion monitoring was applied (107, 121, 135, 149 *m*/*z*—NPs, 113 *m*/*z*—internal standard (^13^C_6_ 4-n-nonylphenol)). 4-Nonylphenols were quantified using an internal standard method. At present, certified reference materials for 4-NPs in both sediments and mussels are not commercially available. All samples were blank- and recovery-corrected. Recovery rates of 4-NPs based on spiking experiments were 94 ± 3 and 83.5 ± 0.4 % for sediments and mussels, respectively. The limits of detection were as follows: 0.25 ng g^−1^ dry weight (d.w.) for a sediment sample and 10 ng g^−1^ d.w. for a mussel sample. The precision of the 4-NP analyses by GC/MS was assessed from the relative standard deviation, which ranged from 2.5 to 5.5 %.

The analysis of 4-NPs in sediments collected in 2008 was not possible because of the unsuitable containers used for their storage.

### Organotin determinations

TBT and TPhT, as well as their derivatives dibutyltin (DBT), monobutyltin (MBT), diphenyltin (DPhT) and monophenyltin (MPhT), were analyzed in the samples according to the procedure described by Pellizzato et al. (2004) and Filipkowska et al. (2011, 2014).

The samples (0.5–2.0 g) were sonication-extracted with a methanol solution of tropolone with the addition of 37 % HCl, centrifuged, and then liquid/liquid partitioned in the extract/dichloromethane/NaCl solution (10 %) system. Next, derivatization with a Grignard reagent (2.0 M pentylmagnesium chloride solution in tetrahydrofuran) and liquid/liquid extraction in the extract/n-hexane/H_2_SO_4_ solution (1 M) system was carried out. The extracts with added internal standard (tripropylpentyltin—TPrT) were reduced under a gentle stream of argon and purified on a column containing activated silica gel (sediment samples) or florisil (mussel samples) soaked with a mixture of n-hexane and toluene (1:1 (*v*/*v*)). The extracts, reduced to 1 mL, were then injected into a gas chromatographic system coupled with a mass spectrometric detector (Varian GC 3900/Saturn 2100T, USA, or Shimadzu GCMS-QP2010 Ultra, Japan). OT concentrations were determined on the basis of response factors derived from daily repeated injections of a standard mixture of derivatized compounds. The complete procedure is presented in detail in the cited papers. The method was validated on the basis of reference materials (CRM 477 (mussels) and BCR 646 (sediments)) and spiked samples (Table 2).

**Table 2:** Recovery yields of the OT procedure, its precision, and limits of detection

TBT	DBT	MBT	MPhT	DPhT	TPhT
*Mussels*					
Recovery based on CE477 [%] *n* = 4					
73	78	96	–	–	–
Recovery based on spiked samples [%] *n* = 3					
59	84	58	30	69	70
LOD [ng Sn g^−1^, d.w.]					
0.6	1.4	0.9	0.3	0.7	0.3
*Sediments*					
Recovery based on BCR646 [%] *n* = 4					
69	68	71	43	75	78
Recovery based on spiked samples [%] *n* = 4					
84	82	59	31	77	66
LOD [ng Sn g^−1^, d.w.]					
0.2	0.3	0.2	0.1	0.2	0.1
Precision [%] *n* = 4					
9	10	9	14	13	15

### Statistical analysis

The results were statistically analyzed using STATISTICA 9.0 software (StatSoft, Poland). The following methods were applied: Shapiro–Wilk normality test, Pearson correlation analysis, R-Spearman correlation analysis, non-parametric ANOVA Kruskal–Wallis, and Mann–Whitney *U* tests. The correlations and differences were regarded as significant for *p* < 0.05.

## Results and discussion

### 4-Nonylphenols

**Fig. 2:**
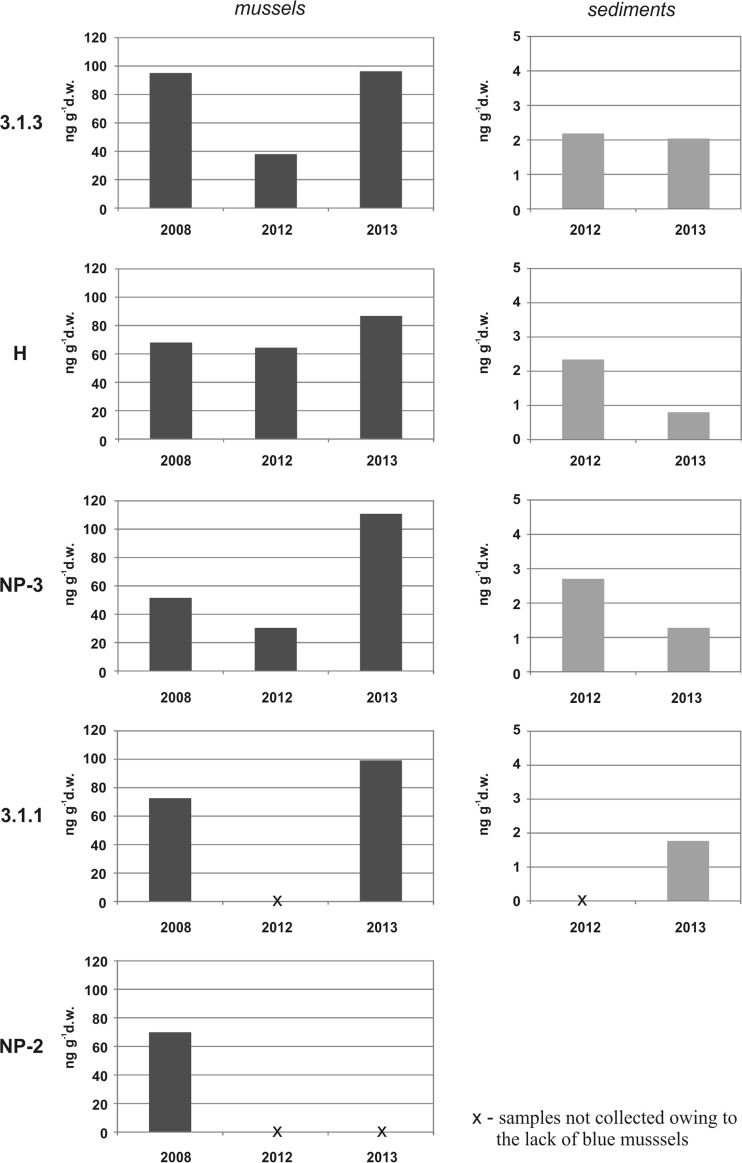
Concentrations of 4-NPs in mussel and sediment samples collected from the Gulf of Gdańsk

4-NP concentrations in mussels ranged from 30 to 111 ng g^−1^ d.w. (mean 74 ng g^−1^ d.w.) (Fig. 2). At each sampling site, the mean 4-NP levels in mussels for the period 2008–2013 were similar and, moreover, there were no statistically significant differences between particular stations (*p* > 0.05, Kruskal–Wallis test). However, temporal variability was observed for mussels collected during the three sampling campaigns (*p* < 0.05, Kruskal–Wallis test). The highest 4-NP concentrations were generally found in mussels sampled in 2013, although the difference between sampling seasons 2008 and 2013 was not statistically significant. The occurrence of 4-NPs demonstrates the exposure of mussels to these contaminants in the western Gulf of Gdańsk and indicates the ability of these bivalves to bioaccumulate 4-NPs. Concentrations of 4-NPs found in all the samples were distinctly lower than the quality standards recommended for biota tissues (10 μg g^−1^ wet weight (w.w.)) to protect predators (secondary poisoning) within the framework of the Common Implementation Strategy for the Water Framework Directive (EU 2005). The observed 4-NP concentrations in *Mytilus trossulus* were similar to those reported by Staniszewska et al. (2014) for samples collected at other stations of the Gulf of Gdańsk in 2011 (Table 3). Similar ranges of 4-NP levels were also found for the Thermaikos Gulf in the northern Aegean Sea and along the coast of Cambodia (Table 3). Nevertheless, the mussels studied in our work generally contained higher 4-NP levels than those sampled from the Thau Lagoon and the North Sea, where a decrease in the concentration of 4-NPs (from 4 to 1.1 ng g^−1^ w.w. (Günther et al. 2001)) in wild blue mussels from 1985 to 1995 was noted. *Mytilus trossulus* from the Gulf of Gdańsk appears to be less contaminated with 4-NPs than mussels collected from many locations worldwide (Table 3). It is worth noting that mussels taken from a farm in NW Spain contained conspicuously higher levels of 4-NPs (380 ng g^−1^ d.w. (Marigómez et al. 2013)).

**Table 3:** Recorded 4-NP and OT concentrations in mussels from different parts of the world

Location	Mussels	Date of sampling	Concentration range				References
*Nonylphenols [ng g* ^*−1*^ *]*							
Southern Baltic Sea:	*Mytilus trossulus*	2008–2013	30–111^b^				This work
Gulf of Gdańsk							
Southern Baltic Sea:	*Mytilus edulis*	2011	19–76^b^				Staniszewska et al. 2014
Gulf of Gdańsk	* trossulus*						
North Sea, Germany	*Mytilus edulis*	1985–1995	1.1–4^a^				Günther et al. 2001
Atlantic Ocean, Spain:	*Mytilus*	2007	380^b^				Marigómez et al. 2013
Coast of Galicia	*galloprovincialis* (farm mussels)						
Mediterranean Sea, France:	*Mytilus galloprovincialis*	2004	32–42^b^				Hong et al. 2009
Thau Lagoon							
Adriatic Sea, Italy:	*Mytilus*	2001–2002	115–240^b^				Pojana et al. 2007
Venice Lagoon	*galloprovincialis*						
Aegean Sea, Greece:	*Mytilus*	2005–2006	27–79^b^				Arditsoglou and Voutsa 2012
Thermaikos Gulf	*galloprovincialis*						
Pacific Ocean, Korea:	*Mytilus*	2006	51–289^b^				Hong et al. 2009
Masan Bay	*galloprovincialis*						
South China Sea:	*Perna viridis*	1995–1999	27–92^b^				Isobe et al. 2007
Cambodian coast							
Java Sea:	*Perna viridis*	1995–1999	75–643^b^				Isobe et al. 2007
Indonesian coast							
Pacific Ocean, USA:	*Mytilus*	2010	Mean 660^b^				Diehl et al. 2012
California, Morro Bay	*californianus*						
Pacific Ocean, USA:	*Mytilus spp*.	2009–2010	96–3000^b^				Maruya et al. 2014
Coast of California			Mean 470^b^				
*Organotins [ng Sn g* ^*−1*^ *]*			TBT	DBT	MBT	∑PhTs	
Southern Baltic Sea:	*Mytilus trossulus*	2008–2013	11–103^b^	5–61^b^	Max. 48^b^	<LOD	This work
Gulf of Gdańsk							
Southern Baltic Sea:	*Mytilus edulis*	1998					Albalat et al. 2002
Gulf of Gdańsk			10.3–38.9^a^	2.2–24.0^a^	1.2–4.7^a^	–	
Pomeranian Bay			15.8–18.1^a^	8.3–8.6^a^	1.9–2.6^a^	–	
Open sea			2.2–12.2^a^	0.5–4.8^a^	Max. 1.5^a^	–	
Southern Baltic Sea:	*Mytilus trossulus*	2003	Max. 133^a^	Max. 37^a^	–	–	Galassi et al. 2008
Gulf of Gdańsk	*Mya arenaria*		149–1 824^a^	Max. 184^a^	–	–	
Atlantic Ocean:	*Mytilus*	2000	11–789^b^	Max. 345^b^	Max. 605^b^	Max. 16^b^	Barroso et al. 2004
Portuguese coast	*galloprovincialis*						
Adriatic Sea:	*Mytilus*	2001–2002	340–7 900^b^	Max. 2 200^b^	Max. 600^b^	Max. 70^b^	Ščančar et al. 2007
Slovenian coast	*galloprovincialis*						
Aegean Sea:	*Mytilus*	2001–2003	Max. 24^a,c^	Max. 16^a,c^	Max. 22^a,c^	Max. 68^a,c^	Chandrinou et al. 2007
Greek coast	*galloprovinciallis*						
Pacific Ocean, Japan:	*Mytilus*	2005	1–118^a,c^	1–47^a,c^	3–22^a,c^	Max. 29^a,c^	Harino et al. 2007
Otsuchi Bay, ports	*galloprovincialis*						
Taiwan:	*Perna viridis*	2003–2004	87–4 329^b^	27–1 015^b^	31–1 314^b^	–	Tang et al. 2010
Luermen Stream estuary							
Mediterranean Sea,	*Mytilus*	1999–2000	Max. 45^b^	Max. 17^b^	Max. 50^b^	–	Mzoughi et al. 2005
Tunisia:	*galloprovincialis*						
Bizerte lagoon							
Pacific Ocean, Canada:	*Mytilus trossulus*	1999	Max. 71^a,c^	Max. 115^a,c^	Max. 61^a,c^	–	Horiguchi et al. 2004
around Vancouver	*Tresus capax*		Max. 914^a,c^	–	–	–	
Atlantic Ocean, USA:	*Mytilus edulis*	1989	Max. 783^b,c^	–	–	–	Peven et al. 1996
Around New York							

*LOD* limit of detection, *TBT* tributyltin, *DBT* dibutyltin, *MBT* monobutyltin, *PhTs* phenyltins

^a^Wet weight basis

^b^Dry weight basis

^c^Values converted into ng Sn g^−1^

The concentrations of 4-NPs in sediments (Fig. 2) were distinctly lower in comparison with mussels and varied from 0.8 to 2.7 ng g^−1^ d.w. (mean 1.9 ng g^−1^ d.w.). Slightly higher, but statistically different (*p* < 0.05, Mann–Whitney *U* test) 4-NP levels were found in 2012 compared to 2013. There were no statistically significant differences between the sediments collected at particular stations (*p* > 0.05, Kruskal–Wallis test). 4-NP concentrations were very low, although all the sampling stations were located near possible pollution sources, i.e., in the vicinity of the Tricity Agglomeration and not far from the mouth of the Vistula River (its catchment area is inhabited by ∼27 % of the Baltic catchment area population). Sediments are considered to be the ultimate sink for hydrophobic pollutants, but many factors influence the distribution of pollutants in the sedimentary matrix. The predominance of a sandy fraction and the low organic matter content in the sediments studied here indicate that the environmental conditions (e.g., hydrodynamic conditions, seabed topography) do not favor a high accumulation of fine particles and their associated 4-NPs in this area. Besides, the high dissolved oxygen content in the near-bottom water may enhance biodegradation and/or accumulation in biota, which results in the decrease of contamination level of sediments. At each station, 4-NP concentrations were much lower than the predicted no effect concentration (PNEC) which was assessed to be 180 ng g^−1^ d.w. or 39 ng g^−1^ w.w.; this threshold was established for freshwater and saltwater sediments as a tentative guideline proposed within the framework of the Common Implementation Strategy for the Water Framework Directive to protect benthic communities (EU 2005). The concentrations of 4-NPs in the sediments studied in this work are comparable to the values reported by Koniecko et al. (2014) for coastal stations of the Gulf of Gdańsk (mean 2.35 ng g^−1^ d.w.), but distinctly higher 4-NP levels were found in surface sediments at some open water stations of the western (up to 249 ng g^−1^ d.w. (Koniecko et al. 2014)) and northern parts of the Gulf of Gdańsk, i.e., Gdańsk Deep (up to 61 ng g^−1^ d.w. (Lubecki 2014)). The sediment samples examined in this work were considerably less contaminated with 4-NPs than those collected from many coastal areas worldwide, e.g., Venice Lagoon (47–192 ng g^−1^ d.w. (Pojana et al. 2007)), Thau Lagoon in France (<2.8–70 ng g^−1^ d.w. (Hong et al. 2009)), Savannah River Estuary in the USA (<0.5–78 ng g^−1^ d.w. (Kumar et al. 2008)), Masan Bay in Korea (24–504 ng g^−1^ d.w. (Hong et al. 2009)), and the northeast coast of China (9–1000 ng g^−1^ d.w. (Wang et al. 2010)). Nevertheless, it should be emphasized that various analytical techniques were used for quantification of 4-NPs in the studies mentioned above.

4-NP concentrations were found to be about 10 to 100 times higher in the mussels than in the sediments. There was a high negative correlation between 4-NP concentrations in mussels and sediments, but this relationship was not statistically significant (*r* = −0.7, *p* > 0.05). This surprising finding may have resulted from the very low 4-NP levels in the sedimentary matrix (∼1–3 ng g^−1^ d.w).

**Fig. 3:**
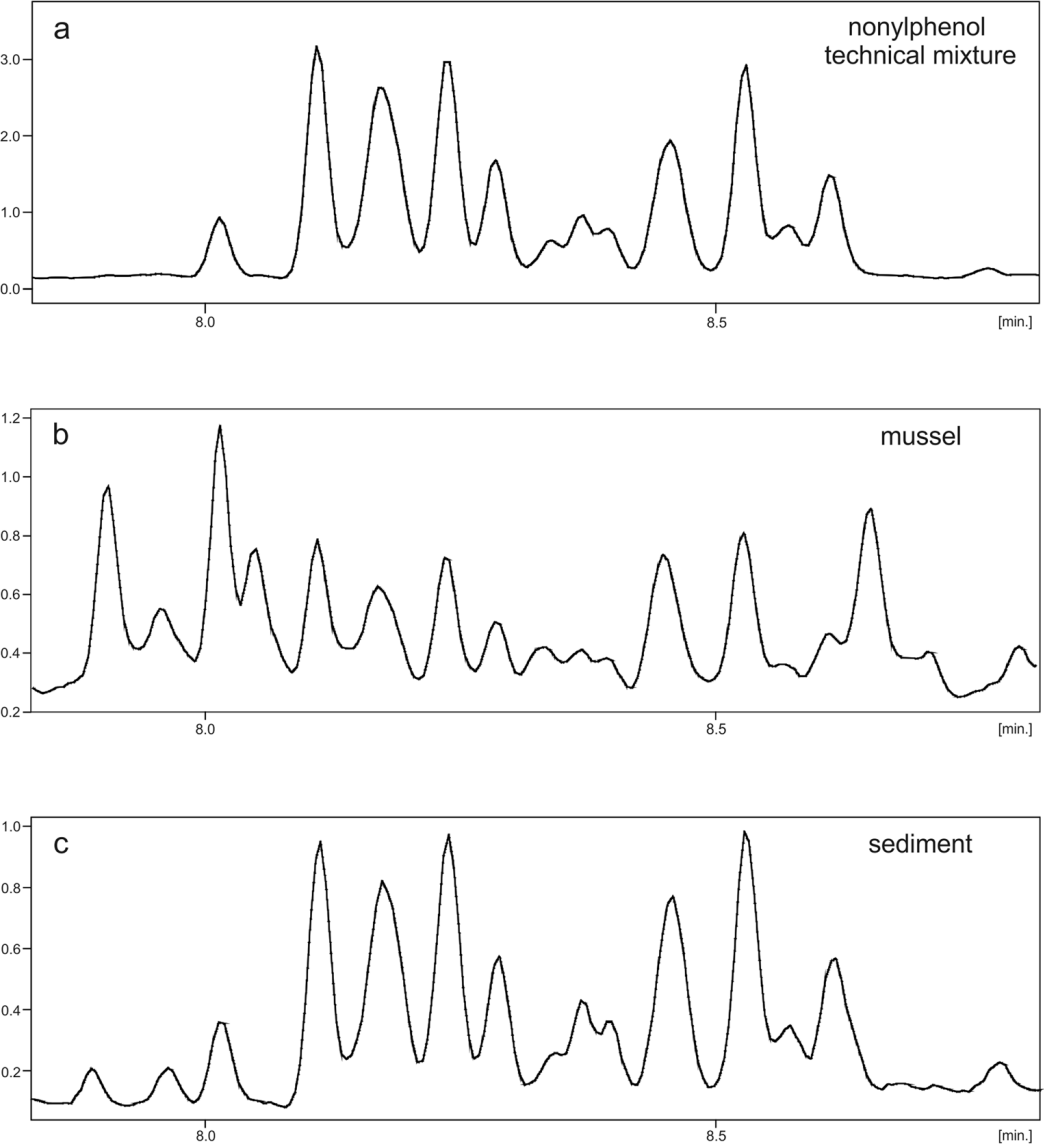
Examples of GC/MS chromatograms obtained from **a** the nonylphenol technical mixture and extracts from **b** mussels and **c** sediments

Relative abundances of particular 4-NP isomer groups in the GC/MS chromatogram (composition patterns) were generally very similar in all the sediments and in the standard (nonylphenol technical mixture). However, there was a greater difference between the 4-NP profiles in mussels and the standard (Fig. 3). This may indicate that 4-NPs and/or their parent compounds (nonylphenol ethoxylates) accumulated in mussel tissues were subject to more significant fractionation/transformation than in the sediments.

### Organotin compounds

**Fig. 4:**
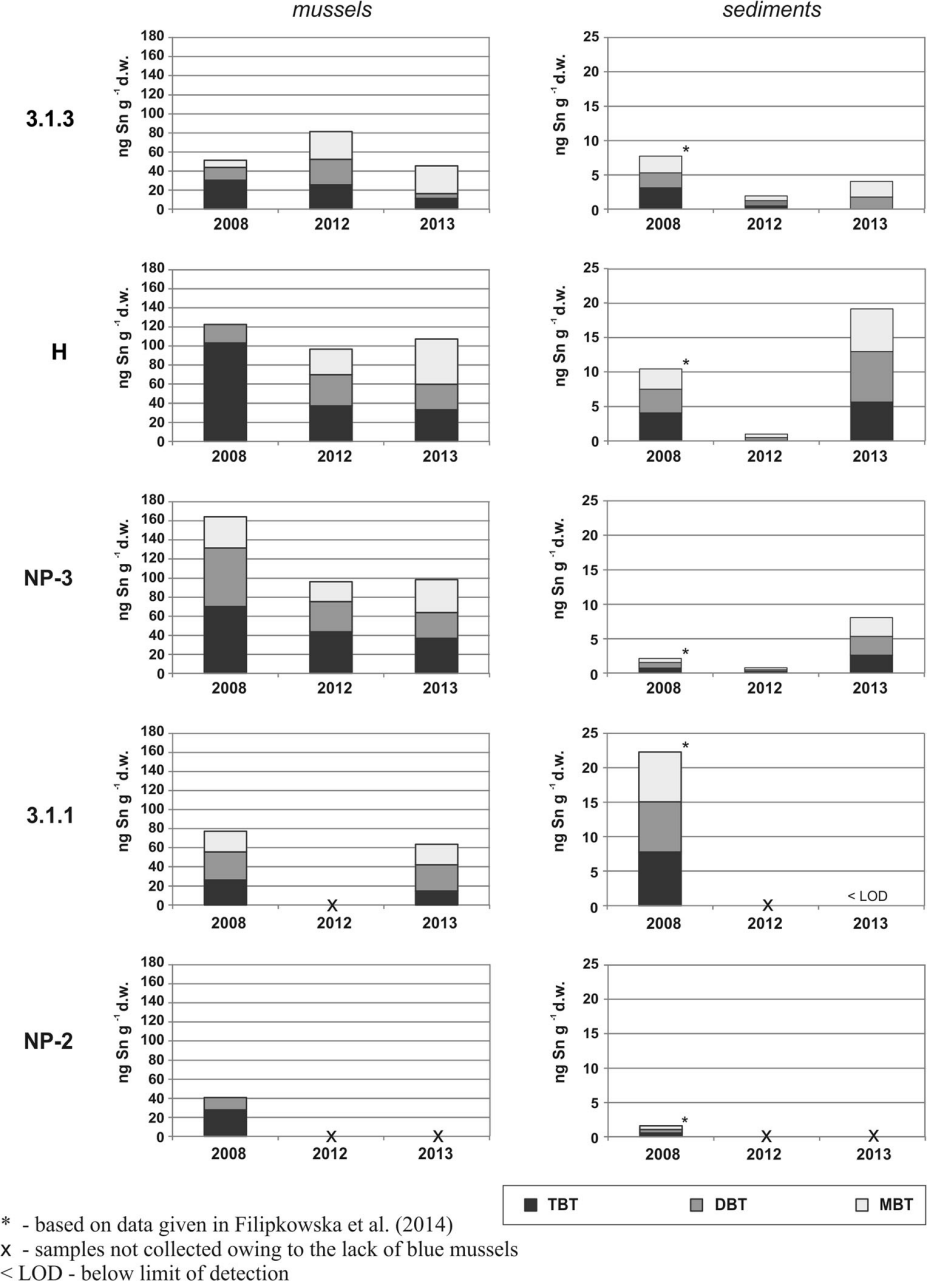
Concentrations of BTs in mussel and sediment samples collected from the Gulf of Gdańsk

Concentrations of OTs in the mussel samples ranged between 41 and 164 ng Sn g^−1^ d.w., while in the sediment samples the highest OT content was 22 ng Sn g^−1^ d.w. (Fig. 4). Only butyltins (BTs) (TBT and its degradation products (DBT and MBT)) were found in both the mussel and sediment samples, whereas phenyltins (TPhT, DPhT, MPhT) were below the limit of detection (LOD).

The results obtained for mussels (Fig. 4) prove that although butyltin compounds are still present in the coastal waters of the Gulf of Gdańsk, their concentrations in the soft tissues of these organisms seem to be relatively low compared with those obtained by researchers in the Southern Baltic Sea and elsewhere in the world (Table 3). However, direct comparison is difficult as different analytical methods are applied and some results are expressed as concentration values per wet weight. Besides, the available data on OTs in mussels concern the period before 2008, when the total ban on using OTs in antifouling paints came into force. As far as the composition of butyltin compounds in mussels is concerned, the mean percentage of TBT in the total butyltin concentration was 43 %, while the respective values for DBT and MBT were 30 and 27 %. This means that in the majority of the samples, TBT > DBT > MBT, which is explained by the limited capability of these organisms to metabolize TBT coupled with their ability to accumulate it (Bortoli et al. 2003; Roper et al. 2001). The slow metabolism of the mussels is also revealed by the values of TBT/∑BTs and TBT/DBT ratios, which are comparable with those obtained in previous studies on marine bivalve mollusks (Table 4). Furthermore, the declining trend for these ratios in the Gulf of Gdańsk over the last few years is worth noting (statistically significant differences for TBT/∑BTs ratio (*p* < 0.05, Kruskal–Wallis test)): it may be explained by the lack of “fresh” input of TBT and the advanced degradation of butyltin compounds in the environment under study.

**Table 4:** TBT degradation indices for the genera *Mytilus* recorded in different parts of the world

Location	Species	TBT degradation indices		References
		TBT/∑ BTs	TBT/DBT	
Gulf of Gdańsk	*Mytilus trossulus*	0.3–0.8^a^	0.9–5.3^a^	This study
		0.3–0.5^b^	0.9–1.4^b^	
		0.2–0.4^c^	0.5–2.1^c^	
Gulf of Gdańsk	*Mytilus edulis*	–	1.6–4.7	Albalat et al. 2002
Venice Lagoon	*Mytilus galloprovincialis*	0.4–0.8	–	Bortoli et al. 2003
Atlantic Ocean	*Mytilus galloprovincialis*	0.4–1.0	1.2–4.0	Barroso et al. 2004
Kattegat	*Mytilus edulis*	–	3.1–14.5	Bellas et al. 2007
Otsuchi Bay	*Mytilus galloprovincialis*	0.2–0.6	0.8–2.5	Harino et al. 2007
Adriatic Sea	*Mytilus galloprovincialis*	0.4–0.9	0.8–16.7	Ščančar et al. 2007

^a^2008

^b^2012

^c^2013

Concentrations of butyltins in the sediments (Fig. 4) were also relatively low: according to the classification suggested by Dowson et al. (1993), the samples were ranked at best as lightly contaminated with TBT (<8 ng Sn g^−1^ d.w.). Moreover, based on values of the butyltin degradation index (BDI) (defined as the ratio between the sum of concentrations of the two main degradation products, DBT and MBT, and that of the parent compound, TBT (Díez et al. 2002)), which were higher than 1 for all the sediment samples, it can be stated that there has not been any fresh input of TBT into the sediments of this region.

In all cases, concentrations of butyltins were at higher levels in mussels than in sediments, which concurs with the results published by Bortoli et al. (2003), Falandysz et al. (2006), Galassi et al. (2008), and Wade et al. (1990). In spite of the fact that these organisms live on the seabed, no statistically significant correlations between OT content in mussels and sediments were found (*p* > 0.05). Being filter feeders, mussels extract particles suspended in the surrounding water, including hydrophobic xenobiotics, which are then accumulated in their tissues. In mollusks, the bioconcentration factor (BCF) of TBT has been reported to range from 1000 to 60,000, and bivalve species are regarded as animals capable of accumulating far larger quantities of butyltins than many other marine organisms (Cao et al. 2009). *Mytilus trossulus* is an epifaunal species for which OTs are available only from seawater, after their remobilization from sediments to the water phase or before these harmful xenobiotics are deposited in sediments. Infaunal species, like *Mya arenaria*, exhibit even a strong ability to extract OTs from bottom sediments and selectively bioaccumulate these compounds (Cao et al. 2009; Galassi et al. 2008; Harino et al. 2005), but as opposed to *Mytilus trossulus*, they live buried in the sediments. The lack of correlations between the OT content in mussels and sediments indicates that remobilization process was not the dominant source of butyltins for mussels from the coastal waters of the Gulf of Gdańsk. However, it is worth considering that OTs may be released from sediments at different rates, despite the fact that both type of sediments (sand) and the environmental conditions at the stations seem to be similar (Table 1). The ratio of butyltin levels in the mussel and sediment samples ranged from 5 to 120; the highest value was recorded at station NP-3 in each campaign. It is worth noting that this station is located close to anchorage of the Port of Gdańsk, where sediments are consistently mixed by the dropping and raising of anchors, which promotes OT remobilization from sediments to the water phase.

As no statistically significant differences between the three campaigns (2008, 2012, 2013) were found (*p* > 0.05, Kruskal–Wallis test) in either the mussel or the sediment samples, we can state that OT pollution in the Gulf of Gdańsk has remained at the same level for the 5 years since the total ban on using OTs in antifouling paints came into force. This period was clearly too short for any changes to manifest themselves, even though favorable conditions for OT degradation prevailed near the coastline of the Gulf of Gdańsk (Filipkowska et al. 2014).

## Conclusions

This study assesses the endocrine disruptor (4-NPs and OTs) contamination of mussels and sediments around the coast of the Gulf of Gdańsk within the last few years. 4-Nonylphenols were found in all the mussel and sediment samples. Butyltins were also found in all the samples, except for one (station 3.1.1, sediments, 2013), while phenyltins were not detected in any of them. No distinct temporal trends for these xenobiotics, either in mussels or in sediments, were observed for the period 2008–2013. The mean concentrations of 4-NPs and butyltins were over ten times higher in mussels than in sediments, but still relatively low compared to worldwide data for both matrices.

Despite the total ban on using harmful OTs in antifouling paints on ships, concentrations of butyltins in the marine environment seem to be at the same level, 5 years after its implementation. In the vast majority of the samples, TBT degradation products (DBT and MBT) were the predominant compounds in the sum of OTs.

Even though the concentrations of 4-NPs and OTs were not alarming in the Gulf of Gdańsk environment, these compounds should be still monitored, as the very presence of these endocrine disruptors in mussels and sediments poses a threat to marine life.
